# Elderly individuals exhibit dysregulated monocyte responses to viral immune complexes compared to adults and children

**DOI:** 10.1038/s41598-025-13883-7

**Published:** 2025-08-01

**Authors:** Léa Domitien Payet, Anthony Coléon, Anne Sophie Bedin, Lucas Auguste, Maël Morvan Duroyon, Caroline Mollevi, Hubert Blain, Franck Mennechet, Éric Jeziorski, Édouard Tuaillon

**Affiliations:** 1 Department of General Pediatrics, Infectiology and Clinical Immunology, Montpellier University Hospital, Arnaud de Villeneuve UHC, 371, Avenue du Doyen Gaston GIRAUD, 34295 Montpellier, France; 2 Pathogenesis and Control of Chronic Infections, INSERM U1058, Montpellier, UHC, University of Montpellier, Montpellier, France; 3 Clinical Research and Epidemiology Unit, CHU Montpellier, University Montpellier, Montpellier, France; 4 France Institute Desbrest of Epidemiology and Public Health, University Montpellier, INSERM, CHU Montpellier, Montpellier, France; 5 Department of Gerontology, Antonin Balmès Center, UHC, Montpellier, France; 6 Virology Laboratory at Montpellier University Hospital, Montpellier University Hospital, University of Montpellier, Montpellier, France

**Keywords:** Immune complexes, Monocytes activation, Immunosenescence, Inflammaging, Innate immunity, Immunology, Infection

## Abstract

The severity of certain viral infectious diseases varies across the age; we hypothesize that these variations could be related to the variation of immune responses to viral immune complexes (ICs) among the age. This study aimed to investigate monocyte activation in response to ICs in children, adults, and elderly individuals. An experimental *in vitro* model was established using peripheral blood mononuclear cells from healthy individuals. Monocyte activation markers (CD169, CD38, HLA-DR), the negative co-stimulatory molecule (PD-L1), and cytokine production were measured under basal conditions and upon stimulation with human adenovirus 5-IgG immune complex (Ad5-ICs), interferon-alpha (IFN-α), and lipopolysaccharide (LPS). Monocytes from children and adults displayed similar activation profiles in response to ICs and IFN-α stimulation, characterized by increased expression of CD169 and PD-L1. In contrast, monocytes from elderly individuals exhibited weak or no overexpression of CD169 and PD-L1 coupled with a diminished PBMC cytokine response. Notably, cells from elderly participants produced high levels of TNF-α, IL-1α, and IL-6 in the absence of stimulation. Multiple comparisons confirmed reduced monocyte activation and PBMC cytokine responses in the elderly compared to adults and children. Although children exhibited a significant response to ICs, their secretion of IFN-α, IP-10, IFN-γ, IL-8, and IL-2 was lower than that observed in adults. Our findings suggest that elderly individuals have poor and dysregulated responses to ICs, likely due to immunosenescence and chronic inflammation. Adults exhibit a robust and balanced response to ICs, while children display a moderate response, possibly influenced by ‘trained immunity’ resulting from frequent early-life exposures to pathogens. These insights highlight the importance of further research to develop age-specific therapeutic strategies to modulate immune function during viral IC exposure.

## Introduction

The heightened susceptibility of older adults to severe outcomes from infectious diseases poses ongoing challenges for public health. Notably, morbidity and mortality related to Covid-19 significantly increase in the elderly population but are rare in children, sparking renewed interest in understanding age-related patterns of infectious diseases^1–3^. Aging is associated with an increase in dysfunction in both innate and adaptive immune responses, resulting in impaired pathogen response and chronic inflammation. This immune impairment associated with aging is termed immunosenescence, and the related inflammation is referred to as inflammaging. Monocytes and macrophages are considered to play a central role in inflammaging^4^. Unlike older adults, children —particularly those of school age— tend to exhibit lower severity in response to many infectious diseases^5^. Thus, school-age children have shown greater resilience than adults, with lower disease severity reported in infections such as influenza, measles, or smallpox^5^.

The control of viral infections heavily relies on the pivotal roles played by type I interferons (IFN) and pro-inflammatory cytokines like TNF-α and IL-6^6^. A balanced and timely IFN-α response is essential for controlling viral infections^7^. During infections like Covid-19, influenza, respiratory syncytial virus (RSV) and dengue, the response of monocytes and macrophages may lead to excessive immune activation^8,9^. Viral infections can induce inflammasome and pyroptosis of monocytes and macrophages^10^. Inflammasome formation begins with the activation of specific cytosolic pattern recognition receptors (PRRs), such as nucleotide-binding oligomerization domain and leucine-rich repeat-containing receptors (NLRs), and AIM2^10^. Fc gamma receptors (FcγRs), receptors for the Fc region of IgG antibodies, are also involved in the activation of monocytes and macrophages^11,12^. AIM2-dependent pyroptosis has been reported in human monocyte-derived dendritic cells in response to ICs formed with human adenovirus^13^. The uptake of SARS-CoV-2 ICs by monocytes and macrophages via FcγRs induces cell death, contributing to systemic inflammation and Covid-19 pathogenesis^12^.

In a previous report, we observed the concomitant presence of high concentrations of both antibodies and SARS-CoV-2 antigen in the blood of adults, children, and the elderly^14–16^. Circulating ICs could potentially serve as an indicator of progression to critical stages in Covid-19^17,18^. Other non-macrophage-tropic viruses, such as measles and RSV, can induce acute respiratory distress syndrome through the involvement of ICs and Fc-mediated responses^19^.

Exploring the distinct features of monocyte responses to ICs in children, adults, and the elderly is crucial for understanding immunopathology and the susceptibility of older adults to infectious threats. It can also contribute to the development of effective therapeutic interventions to mitigate the impact of virus immune complex in these age groups. In this study, we conducted *in vitro* studies to further understand the age-related effects of monocyte activation by ICs.

## Materials and methods (Figure 1A)

**Fig. 1:**
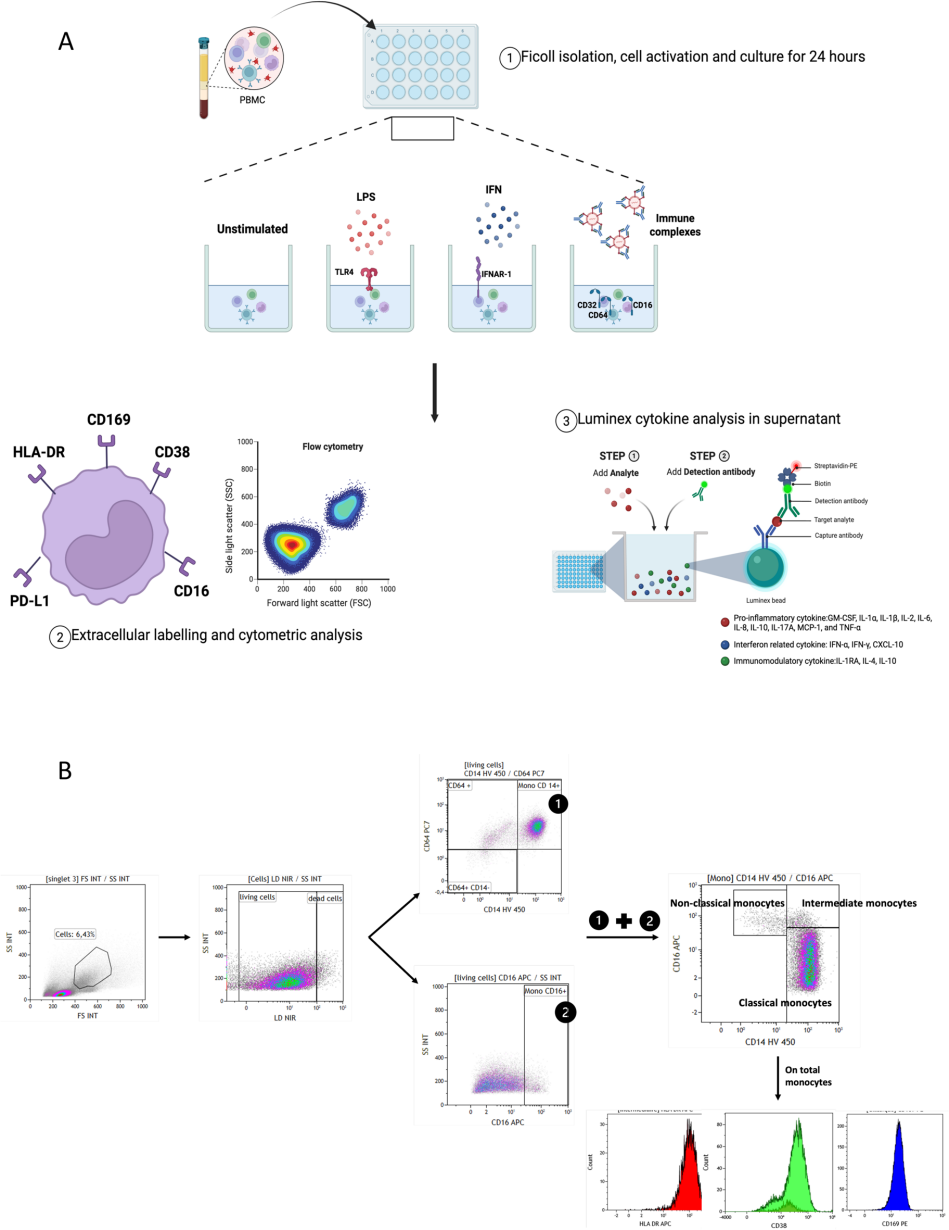
**A**. Overview of Experimental Protocol. **B**. Gatting strategy: PBMCs were first gated based on FSC/SSC to exclude debris and doublets, followed by live cell selection using a viability dye. Monocytes were identified using a composite gating strategy: CD64⁺CD14⁺ and CD16⁺ cells were gated independently within live cells and then combined to define the total monocyte population. Monocyte subsets were classified as classical (CD14⁺⁺CD16⁻), intermediate (CD14⁺⁺CD16⁺), and non-classical (CD14⁺CD16⁺⁺). Expression of activation markers CD169, PD-L1, CD38, and HLA-DR was assessed within the total CD64⁺ monocyte gate.

### Samples

Blood samples were collected from 40 participants who provided informed consent and were confirmed to be healthy and free from fever or viral symptoms in the last 15 days. The inclusion period was between January 2022 and August 2022. Participants were divided into three groups: children under 15 years old, adults aged 15-70, and elderly individuals aged 70 and over. Samples for the children’s group were obtained from endocrinology consultations for growth retardation or puberty screening. These children had no co-morbidities and were not on long-term treatment. Samples in the adult group were collected from French Blood Establishment (EFS) blood donors, and those in the elderly group were obtained from patients consulting for falls or memory disorders. They have no ongoing hematological or tumor pathologies, no chronic or recent infections (within 15 days) and have not received any immunosuppressive treatment. This study was approved by the Montpellier University Hospital Ethics Committee (IRB N°: IRB-MTP_2022_04_202201101). All experimental procedures were performed in accordance with relevant institutional guidelines and regulations, and with the Declaration of Helsinki. Written informed consent was obtained from all participants and/or their legal guardians prior to inclusion in the study.

### Cell culture and stimulation

Fresh PBMCs were isolated using Ficoll density gradient centrifugation and then cultured in RPMI 1640 supplemented with 10% decomplemented fetal calf serum and 1% antibiotics (streptomycin/ampicillin) in a 24-well plate. Each experimental condition was assigned to a separate well, with 1 million cells per well. PBMCs were stimulated with lipopolysaccharide (LPS) (500ng/mL), interferon alpha (IFNα) (400IU/mL), adenovirus type 5 immune complexes (Ad5-ICs) (1000 viral particles per cell), or immunoglobulin alone (IV) (2µL). The cells were incubated at 37 °C for 24 hours (Figure 1A).

### Adenovirus vectors and immune complex formation

Adenovirus -galactosidase (Adgal) is a E1/E3-deleted HAd5 vectors and were produced as previously described^20^. We generated IC-HAdVs by mixing the virus (1 x 10^4^ pp/cell) with 2 μl of IVIg (human IgG pooled from between 1,000 and 50,000 donors/batch) (Behring, France, lot P100390460) for 20 min at room temperature. IVIg is used in patients with primary or acquired immune deficiencies as well as autoimmune diseases. The formation of in vitro ICs was confirmed by dynamic light scattering (DLS), which verified the presence of aggregates following incubation of HAd5 with IVIg (Supplementary Figure 1).

### Analysis of monocyte activation

Following 24 hours of activation, the supernatant from each condition was collected for subsequent cytokine analysis. The cells were then washed and labeled with a cell viability marker, the Near-IR Dead Cell Stain Kit, in the dark for 30 minutes. After adding 20uL of Fc Blocking, the cells were labeled with the following antibody panel: HLA-DR FITC (clone B8.12.2), CD169 PE (clone 7-239), PD-L1 PE-CF594 (clone MIH1), CD64 PC7 (clone 10.1), CD38 AF700 (clone HIT2), CD16 APC (clone 3G8), CD14 HV450 (clone MoP-9) and incubated for 20 minutes in the dark at 4°C.

They were subsequently suspended in PBS supplemented with 0.005% EDTA and 2% decomplemented foetal calf serum. Finally, they were fixed with 2% PFA and kept at 4 °C until analysis (Figure 1A).

The monocytes were analyzed using a Navios flow cytometer with three lasers and 10 colors (Beckman Coulter Inc.). The analysis was performed using Kaluza analysis software (version 2.1; Beckman Coulter Inc.) Monocytes were then identified using a composite gating strategy (Figure 1B). First, CD64⁺CD14⁺ cells were gated within the population of live cells. In parallel, CD16⁺ cells were also gated within the same live cells. A final composite gate combining CD64⁺CD14⁺ and CD16⁺ cells allowed identification of the full monocyte population and discrimination of the three main subsets: classical (CD14⁺⁺CD16⁻), intermediate (CD14⁺⁺CD16⁺), and non-classical (CD14⁺CD16⁺⁺) monocytes. Activation markers—including CD169, PD-L1, CD38, and HLA-DR—were subsequently analyzed on the total CD64⁺ monocyte population (Figure 1B).

Enumeration of CD4⁺ and CD8⁺ T cells and monocytes was also performed as part of routine clinical analysis at the CHU de Montpellier using a Navios cytometer (Beckman Coulter), with an antibody panel including CD3, CD4, CD8, CD14, and CD45.

Monocyte activation was assessed through the expression of CD169^21^, CD38 and HLA-DR. The monocyte expression of CD169, a sialoadhesin belonging to the lectin receptor family, is induced by type 1 interferon making it an early marker of viral infections^21–23^. CD38 is a transmembrane glycoprotein and an enzyme involved in the metabolism of nicotinamide adenine dinucleotide (NAD+) and calcium signaling. CD38 expression is upregulated on monocytes during in response to infections and inflammation. HLA-DR as part of the major histocompatibility complex class II (MHC-II) enables monocytes to present antigens to CD4+ T cells, facilitating the activation of adaptive immune responses. Its expression is upregulated in response to inflammatory signals, such as interferon-gamma (IFN-γ). Conversely, reduced HLA-DR expression on monocytes is associated with immunosuppression, as seen in conditions like septic shock, where it reflects immune paralysis^24^. Programmed death-ligand 1 (PD-L1), also known as CD274, is a surface receptor belonging to the immunoglobulin family that acts as a critical immune checkpoint. While PD-L1 is best known for its inhibitory role on T cells - by binding to PD-1 on activated T lymphocytes to suppress their proliferation and cytokine production^25,26^ -, it also plays a regulatory role in innate immune cells.

In monocytes, PD-L1 expression is typically low at baseline but increases upon stimulation by toll-like receptors (TLR)^27^. Notably, in patients with septic shock, PD-L1 is overexpressed on monocytes^28^. This modulation controls the strength of the immune response.

Fluorescence Minus One (FMO) controls were used to define gating thresholds for all key surface markers, including CD169, CD 38, HLA-DR and PD-L1, ensuring accurate discrimination of positive populations and consistency across samples.

### Cytokine response

Cytokine analysis was conducted on culture supernatants 24 hours after activation using a customised 15-plex panel kit (ProcartaPlex™ Immunoassays, Thermo Fisher). The kit analyzed the following cytokines: GM-CSF, IFN-α, IFN-γ, IL-1α, IL-1β, IL-1RA, IL-2, IL-4, IL-6, IL-8, IL-10, IL-17A, IP-10, MCP-1, and TNF-α using a Luminex MagPix instrument (Figure 1A).

### Statistical analysis

Patients’ characteristics were presented using as medians and ranges (min-max) for quantitative variables, and as frequencies and proportions for categorical variables. Non-parametric comparisons between conditions were performed using the Mann-Whitney test, and comparisons between groups (Child, Adult, Elderly) were performed using the Kruskal-Wallis test, followed by Dunn’s multiple comparison post hoc analysis.

An unsupervised algorithm based on a hierarchical clustering was applied to analyze the relationship between cell biomarkers and age categories and only performed on complete data according to different stimulation conditions. The heterogeneity of these signatures was visualized through a heatmap with a dendogram. Euclidean distance calculated based on root sum of squares differences was chosen as the metric for the dissimilarity matrix.

A Spearman correlation matrix was used to assess association between age and monocyte activation in the ICs condition. A dissimilarity percentage based on the absolute difference the correlation values between group was calculated with a threshold level of 0.1 (dissimilarity was found if absolute difference is more than threshold level).

Volcano plots were generated to study the relationship between the p-values of statistical tests and the magnitude of biomarker differences between groups under IC stimulation. The fold change was calculated as the log2 ratio of the median in each group and the Wilcoxon test was used to determine significance. The larger the difference in groups expression, the more extreme the point appears on the X-axis, while higher -log10(p) values indicate greater statistical significance. All statistical tests were two-sided, and p-values < 0.05 were considered as statistically significant. Statistical analyses were performed using Prism, (GraphPrism10 software) and R (version 4.3.1).

## Results

### Monocytes and CD8 T cells in elderly individuals

The number of monocytes did not differ significantly between the groups, with values of 387 cells/µL (60; 590) in the children’s group, 393 cells/µL (208; 846) in the adult group and 357 cells/µL (200; 367) in the elderly group (Table1). In healthy subjects, three monocyte sub-populations were identified based on the level of expression of CD14 and CD16: classical CD14++, CD16- monocytes (80% of monocytes) primarily dedicated to phagocytosis with minimal involvement in inflammation, intermediate CD14++, CD16+ (around 5% of monocytes), which are involved in both phagocytosis and inflammation, and nonclassical CD14-, CD16++ monocytes (5-10%) which play a limited role in phagocytosis but are crucial for inflammation^29^ (Figure 1B). A reduction in intermediate monocytes was observed in the elderly group (p=0.0063) (Table 1). Due to the low numbers of intermediate and non-classical monocytes, the subsequent results are presented for the total monocytes population. The number of CD8 T cells was also significantly lower in the elderly group with a median of 190 cells/µL (164; 794) compared to 426 cells/µL (281; 852) in adult group and 563 cells/µL (180; 1037) in children, (p=0.047) (Table 1).

**Table 1:** Patient characteristics.

		**Child group** **N=15**	**Adult group** **N=15**	**Elderly group** **N=10**	**P value**
**Age**	Median (min; max)	4.5 (1;6)	39 (23;60)	81 (74;87)	0.0001*
**Sex**	n (%)				
Men		8 (53.33)	10 (66.67)	3 (30.00)	0.238°
Women		7 (46.67)	5 (33.33)	7 (70.00)	
**Biology**					
LT	Median (min; max)	1041 (545;3209)	1310 (1013;1706)	817 (545;1645)	
	*Missing*	4	2	3	
LT CD4	Median (min; max)	662 (331;2163)	821 (613;1187)	600 (412;891)	
	*Missing*	4	2	3	
LTCD8	Median (min; max)	563 (180;1037)	426 (281;852)	190 (164;794)	0.047*
	*Missing*	4	2	3	
Monocytes CD14+	Median (min; max)	387 (60;590)	393 (208;846)	357 (200;367)	
	*Missing*	4	1	3	
Classical monocytes CD14+CD16-	Median (%) (min; max)	78.6 (45.09;89.08)	75 (38.07;91.14)	81.6 (56.13;94.54)	
Intermediate monocytes CD14+ CD16+	Median (min; max)	4.7 (0.23;31.14)	5.3 (1.54;27.22)	2.3 (0.77;11.85)	0.006*
No-classical monocytes CD14- CD16+	Median (min; max)	16.2 (2.79;39.16)	19.6 (2.62;49.86)	15.7 (4.3;42.69)	

*Kruskall Wallis test, °Fisher’s exact test. Only significant p-values are reported.

.

### Age-dependent differences in cell surface marker expression after in vitro stimulation

**Fig. 2:**
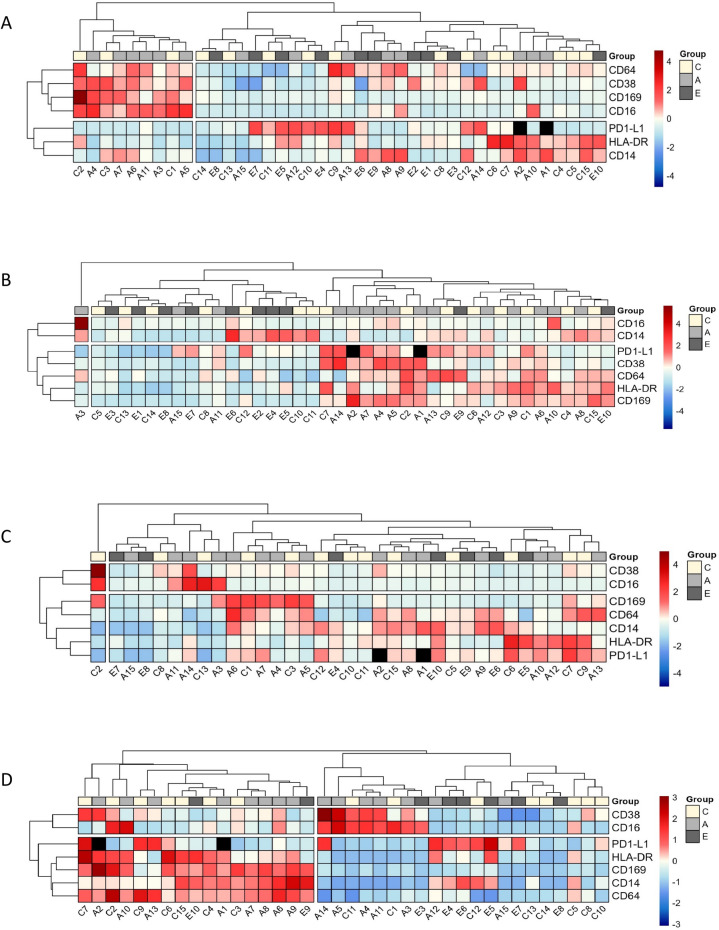
Hierarchical clustering of all biomarkers for no stimulation, on 40 patients (**A**), stimulation with immune complexes, on 40 patients (**B**), stimulation with LPS, on 35 patients (**C**) and stimulation with IFNα, on 38 patients (**D**). Levels are colored from low (dark blue) to high (dark red). C= child group, A= adult group, E= Elderly group.

We first analyzed monocyte activation patterns after stimulation with ICs, IFNα, or LPS in the different age groups using unsupervised hierarchical clustering analysis. Although notable dispersion of participants was observed within each group, visual inspection suggests a differential expression of cell surface marker between elderly individuals and adults/children in both unstimulated cells and cells stimulated by ICs and IFNα (Figure 2). Following ICs stimulation, eight out of ten elderly individuals were grouped in the cluster located on the left part of the heat map (Figure 2B).

**Fig. 3:**
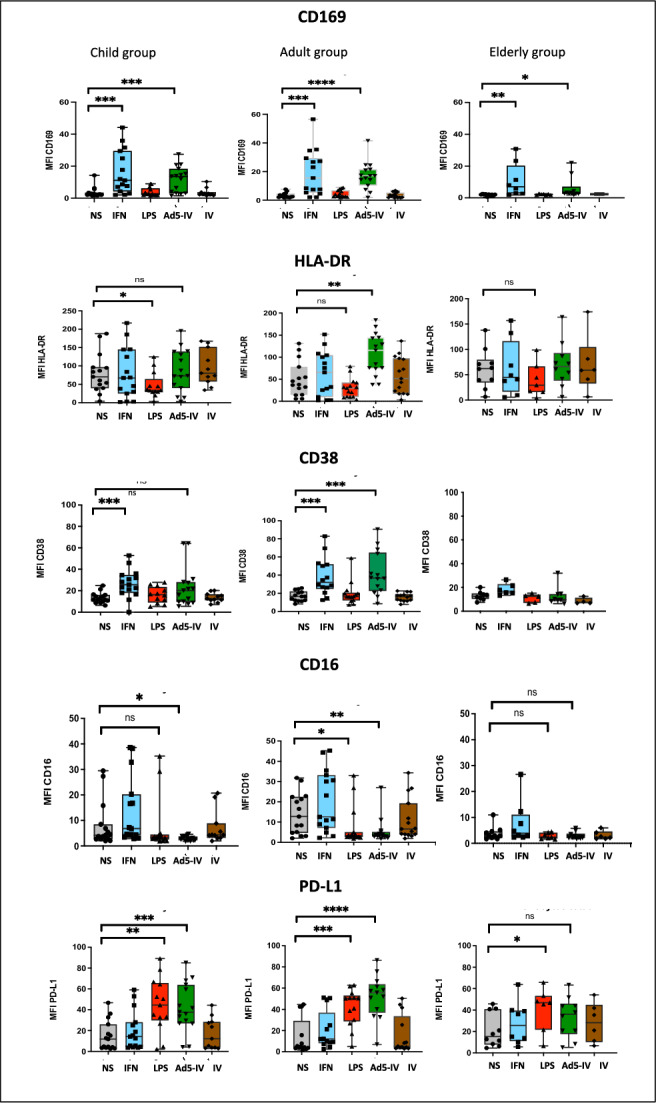
The median fluorescence of CD169, HLA-DR, CD38, CD16 and PD-L1 on total monocytes was measured in child, adult and elderly groups under the following experimental conditions: unstimulated, IFNα 400U/mL, LPS 500ng/mL, Ad5 (1000 particles per cell) + Immunoglobulins (2uL) (Ad5-IV/ICs), and Immunoglobulins alone (IV) (2uL). ****p < 0.0001; *p < 0.05; ns = not significant.

The analysis of activation markers following IC stimulation showed that CD169 expression increased in all three groups compared to the unstimulated condition (Figure 3). However, the response was lower in the elderly group than in adults (p=0.0048) (Table 2, Figure 3). CD38 and HLA-DR expression increased significantly in the presence of ICs in the adult group, while CD16 expression decreased in the child and adult groups (Figure 3). Stimulation of PBMCs with immunoglobulin alone (Figure 3) and in the presence of adenovirus alone (data not shown) did not result in the activation of CD169.

**Table 2:** Biomarkers’ Inter-group statistical comparison.

Stimulation condition	Cell marker	Child group n = 15 *Median* *(min, max)*	Adult group n = 15 *Median* *(min, max)*	Elderly group n = 8 *Median* *(min, max)*	Comparison between child, adult and elderly groups			
					***P value***			
					**K-W**	**Multiple comparisons**		
						**C vs A**	**C vs E**	**A vs E**
NS	CD169	2.51 (1.54; 14.33)	2.67 (1.71; 7.65)	2.14 (0.56; 2.56)	0.09	**-**	**-**	**-**
	CD38	11.60 (6.25; 24.76)	16.53 (0.74; 25.78)	11.47 (0.37; 20.11)	0.10	**-**	**-**	**-**
	PD-L1	11.86 (2.66; 49.69)	4.71 (1.65; 44.62)	15.24 (4.5; 45.72)	0.20	**-**	**-**	**-**
	HLA-DR	70.56 (3.94; 188.07)	37.92 (5.55; 131.08)	62.43 (6.46; 137.86)	0.20	**-**	**-**	**-**
IFN	CD169	11.15 (2.09; 44.6)	15.53 (2.1; 56.58)	6.98 (1.88; 30.82)	0.48	**-**	**-**	**-**
	CD38	25.73 (0.05; 52.85)	32.01 (1.15; 82.84)	15.31 (0.52; 26.48)	**0.03**	0.26	**0.02**	0.10
	PD-L1	14.26 (3.59; 59.11)	12.09 (1.15; 82.84)	25.73 (5.62; 63.82)	0.45	**-**	**-**	**-**
	HLA-DR	62.71 (2.04; 217.10)	65.52 (1.70; 151.81)	44.74 (5.57; 156.96)	0.73	**-**	**-**	**-**
ICs	CD169	13.60 (1.54;27.47)	17.01 (1.86;41.53)	3.05 (1.83;22)	**0.02**	0.26	0.24	**0.048**
	CD38	20.21 (5.4;64.24)	36,12 (0.12;90.57)	9.34 (0.02;32.02)	**0.003**	0.097	0.27	**0.02**
	PD-L1	37.73 (4.24;85.05)	55.67 (6.87;86.24)	36.01 (5.02;63.37)	0.14*	**-**	**-**	**-**
	HLA-DR	87.40 (3.18; 195.21)	115.39 (37.56; 184.10)	66.26 (5.72; 163.77)	0.21	**-**	**-**	**-**
LPS	CD169	2.97 (1.95; 8.87)	2.71 (1.66; 9.00)	2.25 (1.61; 2.72)	**0.03**	**-**	**-**	**-**
	CD38	16.90 (5.20; 124.87)	16.32 (1.11; 58.77)	7.47 (0.53; 15.11)	**0.03**	**-**	**-**	**-**
	PD-L1	44.53 (2.13; 89.22)	50.3 (5.05; 62.8)	46.9 (6.57; 65.80)	0.98	**-**	**-**	**-**
	HLA-DR	33.56 (3.61; 124)	29.95 (3.69;79.08)	29,42 (4.28;98.91)	0.59	**-**	**-**	**-**

K-W=Kruskal Wallis Test, C= Child, A=Adult, E=Elderly.

*With Mann-Whitney test between adult and elderly group in ICs condition, there is a significant difference (p=0.04) but this difference was not found with multiple comparisons.

Activation of monocytes upon IFNα stimulation resulted in an increase of CD169 expression, compared to unstimulated condition (Figure 3), with no significant difference between the three groups (Table 2). CD38 expression also increased in the child and adult groups (Figure 3), with a lower expression in the elderly group (p= 0.003) (Table 2). PD-L1 expression increased after IC stimulation in adults and children, but not in the elderly group (Figure 3) and following LPS activation, an increase was observed in all three group (Figure 3).

### Reduced cytokine response upon stimulation, with elevated basal secretion in cell supernatants from elderly individual

**Fig. 4:**
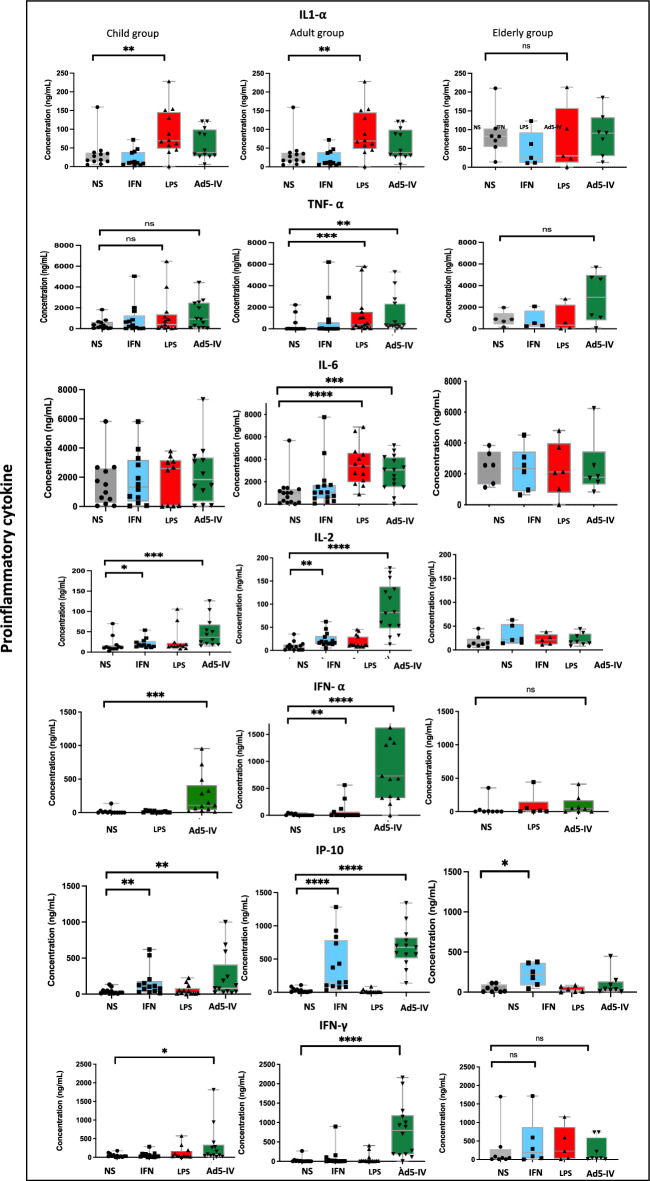

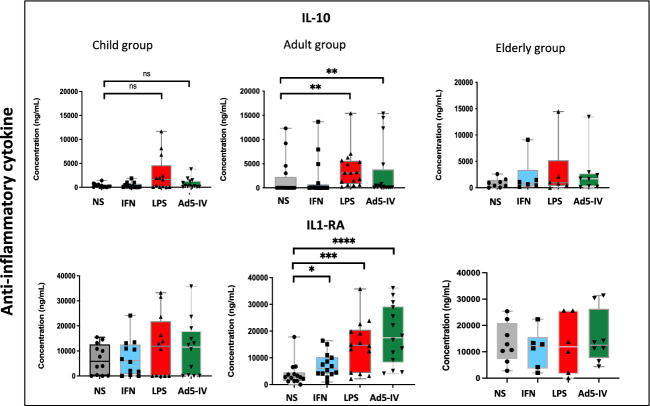
The concentration of IFN-α, IFN-γ, IL1-α, IL1-RA, IL-2, IL-8, IL-10, IP-10 and TNF-α on monocytes’ culture supernatants was measured in child, adult and elderly groups under the following experimental conditions: unstimulated, IFN 400U/mL, LPS 500ng/mL, Ad5 (1000 particles per cell) + Immunoglobulins (2uL), and Immunoglobulins alone (2uL). ****p < 0.0001; *p < 0.05; ns = not significant.

ICs stimulation induced IFN-α and IFN-γ secretion, with a weaker response observed in the elderly group compared to adults (p=0.001, p=0.04, respectively) (Supplementary Table 1). In the adult group, there was increased secretion of pro-inflammatory and Th1 cytokines (TNF-α, IL-1α, IL-2, IL-6 and IL-8), as well as elevated anti-inflammatory cytokines (IL-10 and IL-1RA). By contrast, cytokine secretion did not significantly increase in the elderly individuals. Upon IFN- α stimulation, IP-10 secretion was increased in the three groups, but with no significant difference between them (Supplementary Table 1). Notably, the basal secretion of TNF- α,IL-1 α and IL-6 was higher in elderly compared to the adult group (Supplementary Table 1), suggesting a weaker dynamic response upon IC and IFN-α stimulation, alongside increased inflammatory cytokine secretion in the absence of stimulation (Supplementary Table 1). LPS stimulation induced an increase in IL1- α and IL-6 (p=0.0001 and p<0.0001, respectively), with a trend for IL-8 (p=0.07) in adults, and an increase of TNF- α (p=0.0008) in both children and adults (Figure 4).

### Comparative analysis of monocyte activation markers and cytokine levels under ICs stimulation across age groups

**Fig. 5:**
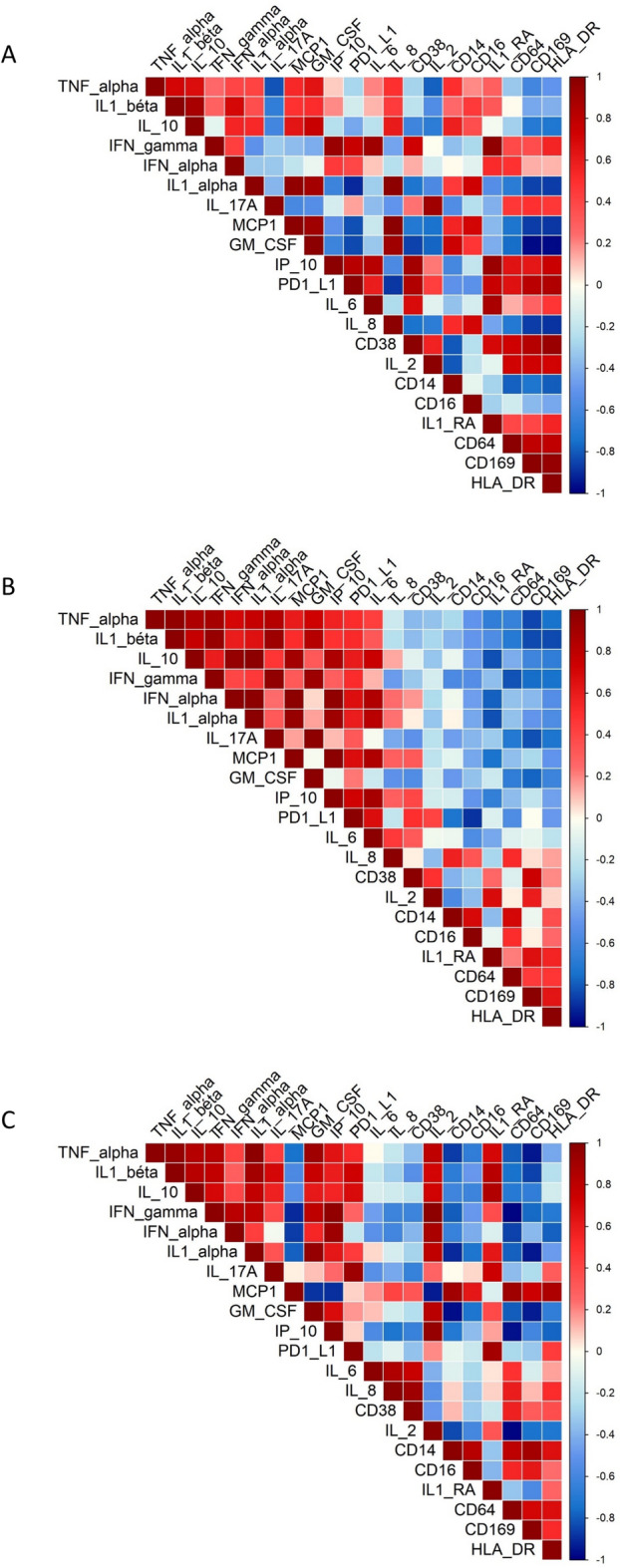
Correlation table of monocyte activation in children (**A**), adults (**B**) and the elderly (**C**) during stimulation by immune complexes.

The correlation profiles under ICs stimulation were examined across the three age groups (Figure 5). The correlation matrix showed greater dissimilarity between the child and elderly groups (13.8% similarity) than between the adult and elderly groups (21.1% similarity) or the child and adult groups (16.1% similarity).

We performed intergroup and coupled comparisons to analyze the differences in monocyte activation markers between children, adults, and the elderly individuals under different stimulation conditions (Table 2). Under ICs stimulation, CD169 expression differed significantly between the three groups (p = 0.02), with coupled comparisons showing higher expression in adults compared to the elderly (p = 0.048) (Table 2). CD38 expression was also significantly different, with higher expression observed in adults compared to the elderly (p = 0.02) (Table 2).

The coupled comparison analysis of cytokine responses under IC stimulation revealed higher IFNα levels in adults than the elderly (p=0.001) and children (p=0.02). Similarly, IP-10 levels were higher in adults compared to children (p=0.04) and the elderly (p=0.005). IFN-γ levels were also higher in adults compared to the elderly (p=0.04). Also, IL-2 levels were lower in the elderly group than in adults (p=0.002), while the TNF-α levels were higher in the elderly than adults (p=0.03) (Supplementary Table 1).

**Fig. 6:**
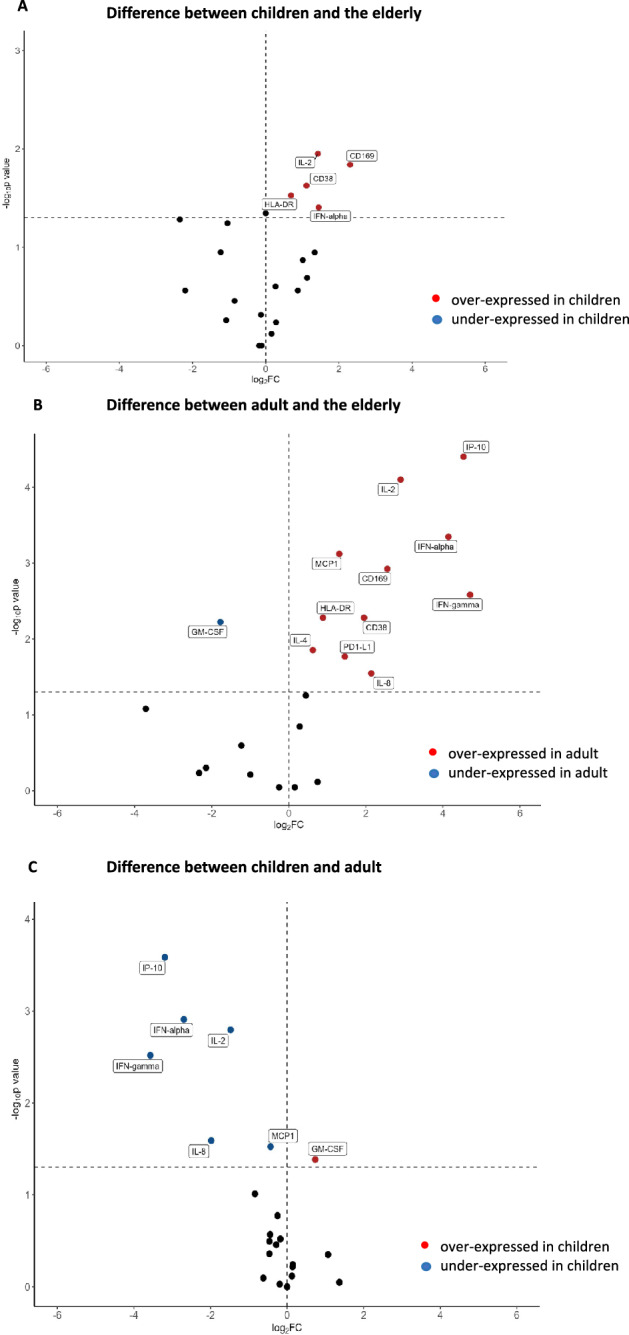
Volcano plots of children versus elderly (**A**) of adult versus elderly (**B**), of children versus elderly (**C**) under stimulation with immune complex, Black circles are not significantly different between two groups. Data were z-scored prior to analysis.

Volcano plots analysis indicated that five out of 22 biomarkers (22.7%)—CD169, CD38, HLA-DR, IL-2, and IFN-α—were significantly different between the child and elderly groups, with all showing higher levels in children compared to the elderly (Figure 6A). When comparing the adult and elderly groups, 11 out of 22 markers (50%) showed significant differences, with the adult group expressing higher levels of CD38, CD169, PD-L1, HLA-DR, IFN-α, IFN-γ, IP-10, IL-8, IL-2, and IL-4 (Figure 6B). Between adults and children, six out of 22 biomarkers (27.2%) differed, with children showing lower expression of IFN-α, IP-10, IFN-γ, IL-8, and IL-2 (Figure 6C).

## Discussion

**Fig. 7:**
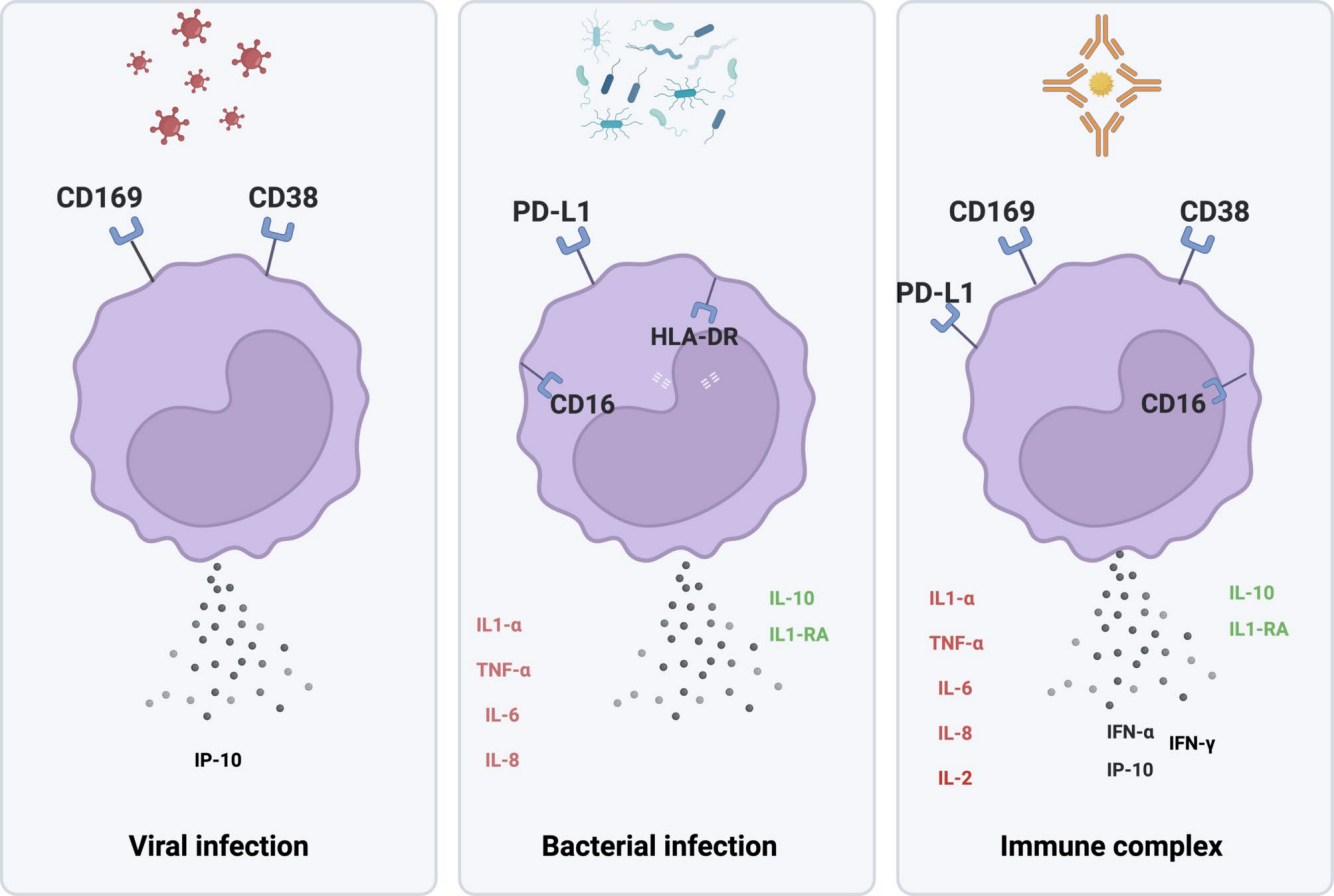
Monocyte activation profile during viral and bacterial infection and in the presence of immune complexes.

**Fig. 8:**
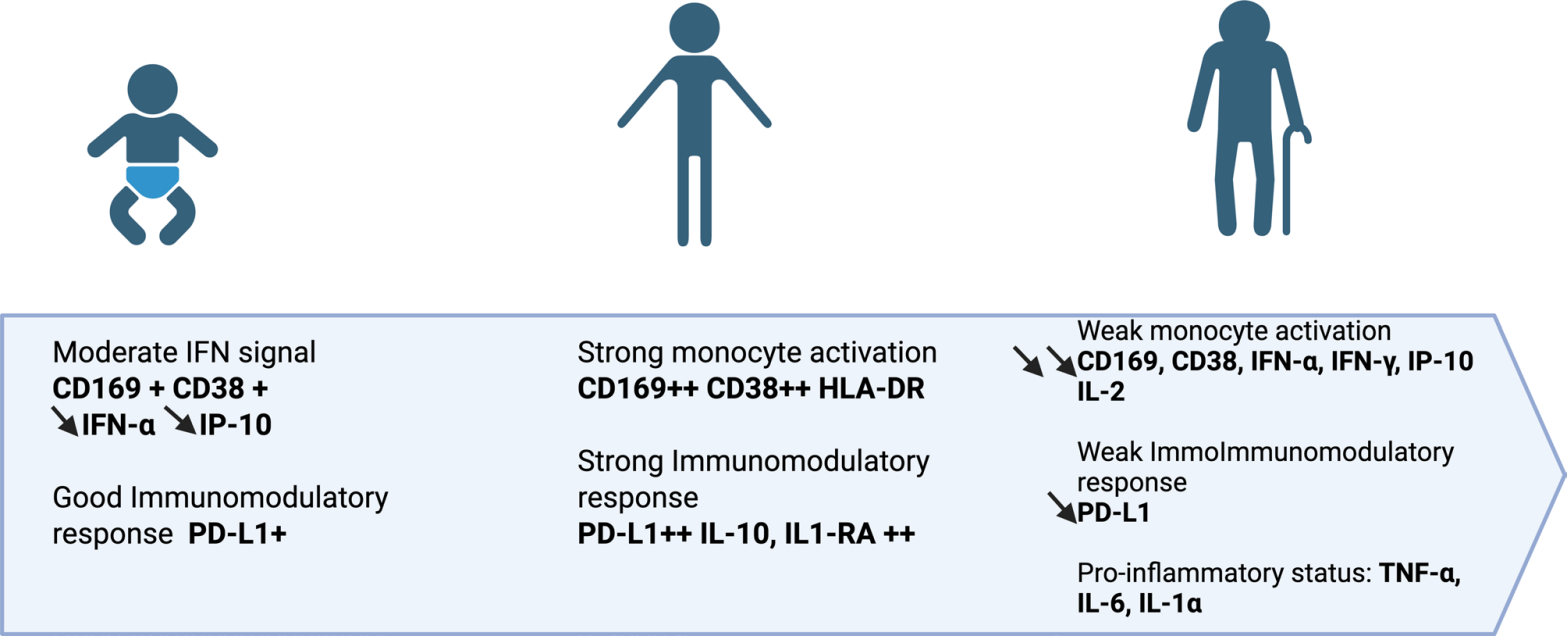
Age-related differences in monocyte activation and its regulation.

In this study, we established an experimental model to characterize monocyte activation in response to viral (IFN-α), bacterial (LPS), and inflammatory (ICs) stimuli (Figure 7). Focusing on ICs, we identified immune profiles that may contribute to age-related susceptibility to severe infectious diseases. Analysis of monocyte activation markers and PBMC cytokine responses revealed distinct, age-dependent immune patterns (Figure 8).

Monocytes from children and adults exhibit comparable activation profile in response to ICs. This profile was characterized by increased CD169 expression and cytokine secretion, although the response was somewhat weaker in children. In adults, the immune response was also more pronounced following LPS stimulation, with increased expression of HLA-DR and CD38, along with elevated IL-6 and TNF-α levels. In contrast, monocytes from elderly individuals displayed a reduced response to ICs activation. Furthermore, when stimulated with IFN-α, CD169 and CD38 expression increased in children and adults but not in elderly individuals, suggesting a diminished capacity of monocytes from elderly individuals to achieve proper activation. We also observed a decrease in CD16 when stimulated by ICs and LPS. CD16 and TLR4 interact with LPS and moved from cell membrane to an intracellular localization^30^. During SARS-CoV-2 infection, monocytes have been shown to uptake virus immune complexes through the Fcγ receptors CD16^12^, suggesting that the decrease in CD16 observed upon ICs stimulation indicates its internalization.

The balanced secretion of pro-inflammatory cytokines such as IL-1α, TNF-α, and IL-6, alongside anti-inflammatory cytokines, including IL-10 and IL1-RA following ICs stimulation, suggests an effective regulation to ensure pathogen elimination and minimize tissue damage. Elderly individuals exhibited a more inflammatory basal state, with higher levels of pro-inflammatory cytokines like TNF-α, IL-1α, and IL-6 compared to adults. This chronic low-grade inflammation, commonly referred to as “inflammaging”, may contribute to increased susceptibility to infection^31^. The altered function of immune cells, including monocytes, is a hallmark of immunosenescence. This is characterized by increased basal cytokine production coupled with a weaker dynamic response, as observed in our study for TNF-α and IL-1α^32^. Such a reduced capacity for cytokine induction could impair the resolution of infections or the clearance of immune complexes, potentially leading to prolonged or unresolved inflammation.

Aging is associated with decreased type I IFN production, linked to fewer circulating dendritic cells and their diminished capacity to produce type I IFNs upon stimulation^33–35^. Since IFN-γ production is strongly regulated by type I IFN, impairments in type I IFN signaling during aging affect also IFN-γ production^36^.

An effective immune response during infectious processes relies on a delicate balance between rapid, robust activation and mobilization of leukocytes, followed by a timely return to a resting state to resolve inflammation. This dynamic involves an initial innate immune response driven by IFN-I, which act as key mediators of early antiviral defense, followed by the adaptive immune response^37^. Importantly, regulatory mechanisms ensure that these responses do not lead to immunopathogenesis^37^. The expression of the CD169 marker on monocytes and macrophages is closely linked to the type I IFN response. CD169, is upregulated on blood monocytes, particularly during viral infections such as Covid-19 in both adults^21^ and children^38^. In the early phase of SARS-CoV-2 infection, people over 65 years had reduced monocyte activation with lower CD169^39^. An impaired type I IFN response is associated with a higher risk of severe influenza A^40^ and SARS-CoV-2^41^ infections. The timing of the type I IFN response during infection has emerged as a key factor for inducing protective versus pathological inflammation. The early response during viral infection protects against severe inflammation, while delayed and dysregulated type I IFN production exacerbate pathology^36^. The transcriptional and functional response of monocytes and macrophages vary with aging, causing alterations in cell activation and decreased production of interferons and cytokines^42^. Notably, RIG-I signaling, a central regulator of type I IFN production, is impaired in older individuals^34,36^. Our results indicate that when monocytes from the elderly are stimulated in vitro by IFN-α, both CD169 expression and IP-10 secretion increased, in contrast to the response observed with IC stimulation. This suggests that, despite intrinsic impairments in signaling pathways, monocytes from older individuals retain some capacity to respond to direct IFN-α stimulation.

The lower expression of PD-L1 in the elderly group compared to children and adults under IC and LPS activation further supports the notion of an impaired immunoregulatory response in this population. PD-L1, a critical checkpoint molecule, plays a key role in modulating immune responses by inhibiting monocyte activation, cytokine secretion and inflammation, thereby regulating the strength of the immune system response. The weak PD-L1 response in the elderly group suggests poorer regulation of the immune response following activation by ICs and LPS. This reduced expression of PD-L1 may contribute to the failure to adequately control inflammatory responses, potentially leading to the chronic low-grade inflammation characteristic of inflammaging^32,43^. Although PD-L1 expression was assessed on monocytes, stimulation was performed on PBMCs, where cell–cell interactions may influence its expression. The increase of PD-L1 on monocytes may reflect not only direct activation but also immune crosstalk within the PBMC environment, including with T lymphocytes.

Comparisons using volcano plot indicated that ICs trigger a stronger Th1 (CD169, IFN- α, IP-10) and inflammatory response (CD38, HLA-DR, IFN- γ, IL-8) in adults compared to elderly individuals. The same pattern was observed in children versus elderly individuals, although the differences were less pronounced. The comparison between children and adults further confirmed that children exhibit a more moderate immune response to ICs. Thus, adults exhibited the most robust response, children a moderate response, and elderly individuals the weakest response. Due to the numerous viral and bacterial infections, as well as vaccinations encountered during early childhood, a child’s innate immunity undergoes a process known as’trained immunity.'^44^ The concept of’trained immunity’suggests that repeated exposure to infections and vaccinations in early childhood not only builds adaptive immunity but also enhances the innate immune response. This enhancement is achieved through epigenetic and transcriptional modifications that improve the responsiveness and functionality of innate immune cells, providing broad-spectrum protection during the early years of life^45^.

Complementary findings on monocyte subsets and T-cell populations provide additional evidence of the cellular changes associated with immunosenescence. We observed a decrease in intermediate monocytes in the elderly compared to adults and children, which contrasts with previous studies reporting an age-associated increase in CD16⁺ monocytes (intermediate and non-classical)^43,46^. This discrepancy may be partly attributable to our gating strategy, which defined monocytes as CD64⁺ cells. Although CD64 is a well-established monocyte marker, and neutrophil contamination is unlikely given the use of Ficoll-isolated PBMCs and the non-inflammatory status of our cohort, a residual overlap with CD16⁺ lymphocytes - particularly NK cells- cannot be fully excluded.

CD16+ monocytes exhibit senescent characteristics, such as shorter telomeres and elevated markers associated with the senescence-associated secretory phenotype (SASP)^47,48^. Furthermore, monocytes from elderly individuals display impaired phagocytosis but show significantly higher intracellular levels of TNF-α both at baseline and following TLR4 stimulation, indicating dysregulated monocyte function with aging^43^. The production of IL-6 and IL-8 have been shown to increase with aging^49^. In addition, we noted a lower number of CD8+ T lymphocytes in elderly individuals. The loss of CD8+ T cells, especially naïve subsets is associated with a state of immunosenescence^50^.

The findings of this study provide insights into age-related differences in monocyte activation and PBMC cytokine responses upon IC stimulation, despite the relatively small sample size. Notably, several significant results consistent with previous literature support the robustness of our observations. This study represents a preliminary step toward ongoing research on larger cohorts. One limitation is the combined analysis of all monocyte subsets rather than examining subpopulations, such as classical, intermediate, or non-classical monocytes, which play diverse roles in immune regulation and inflammation. In addition, the recruitment of older participants from memory and falls clinics may introduce selection bias related to frailty or subclinical inflammation, which should be considered when interpreting age-related findings.

## Conclusion

In conclusion, our study highlights age-related differences in monocyte activation induced by ICs. Adults demonstrated the most robust immune response, characterized by increased expression of activation markers, cytokine production, and immunoregulatory molecules. This balanced response may contribute to their relative resistance to severe viral infections compared to the elderly. Conversely, the elderly exhibited weakened responses, including reduced CD169 and PD-L1 expression and impaired interferon production, likely related to chronic inflammation and immunosenescence. Children showed a more moderate immune response, likely reflecting their ‘trained immunity’ from early-life exposures to infections.

These findings underscore the importance of age as a determinant of immune susceptibility to ICs generated during infections and suggest that therapeutic strategies targeting immune regulation, particularly in the elderly, could help mitigate age-related vulnerability to infections. Further research is needed on larger cohorts and with investigations into the molecular mechanisms driving these age-related differences in immune function.

## Supplementary Information

Supplementary Information.

## Data Availability

All data generated or analyzed during this study are included in this published article and its supplementary information files.
