# Supplementary material for: Elderly individuals exhibit dysregulated monocyte responses to viral immune complexes compared to adults and children
Source: Sci Rep. 2025 Aug 1;15:28186. doi: 10.1038/s41598-025-13883-7 (PMC12316873; doi:10.1038/s41598-025-13883-7)
Supplement: Supplementary file 1 — Supplementary Information. [file 41598_2025_13883_MOESM1_ESM.pdf]

Supplementary data

Supplementary Figure 1: Dynamic Light Scattering (DLS) analysis of in vitro-generated immune complexes using IVIg and HAd5.

DLS is a biophysical technique that measures fluctuations in light scattering caused by Brownian motion of particles in suspension, allowing estimation of their hydrodynamic diameter.

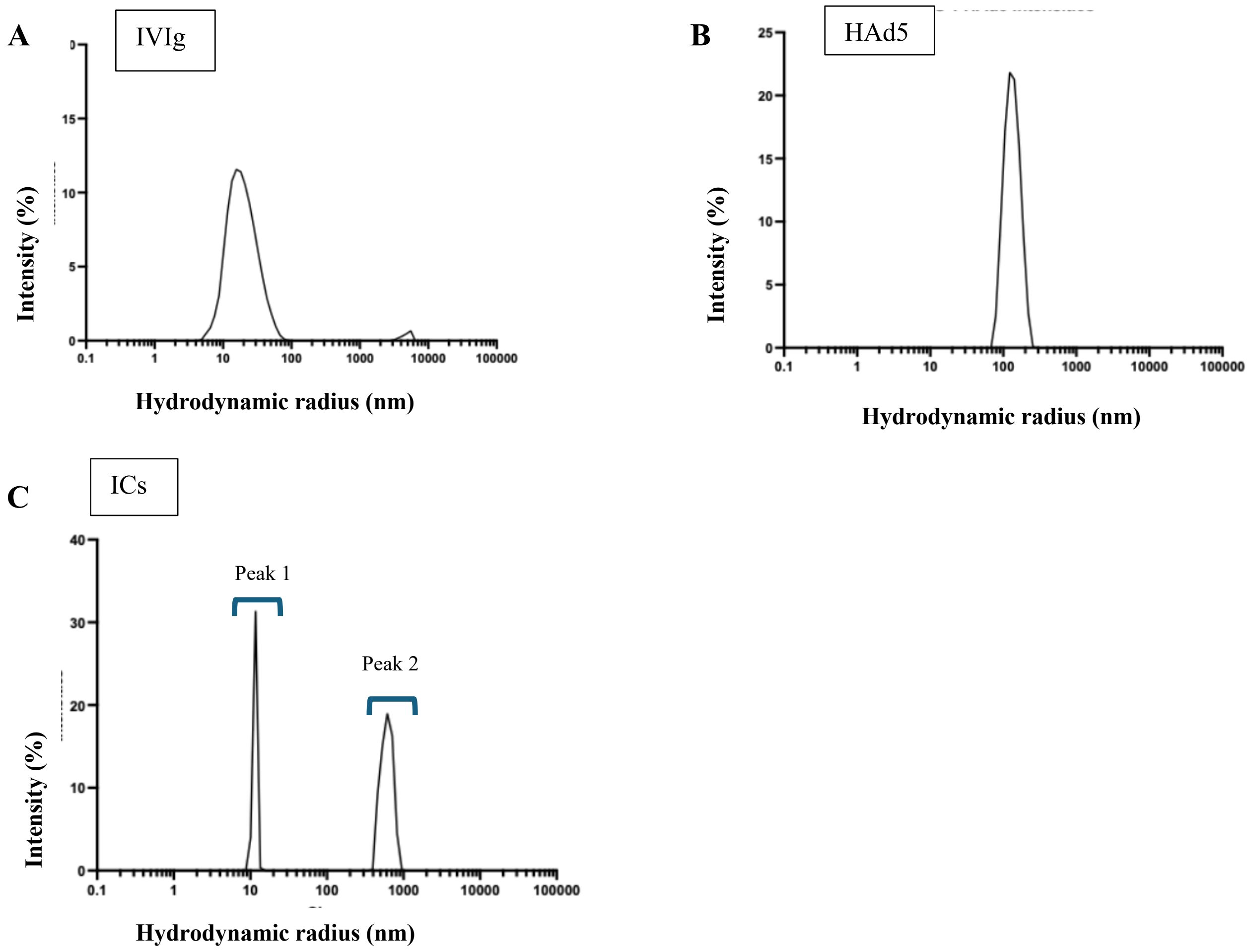

A. The main peak is centered around 10-20nm, corresponds to monomeric or low-order oligomeric IgG molecules.
B. The DLS analysis of the adenovirus preparation showed a major peak around 100 nm, which is consistent with the known hydrodynamic diameter of human adenoviruses. This result confirms the expected size range of 90–100 nm typically reported for non-enveloped icosahedral adenoviral particles.
C. The peak 1, centered around 10–20 nm, corresponds to monomeric or low-order oligomeric IgG molecules. A secondary peak near 1000 nm indicates the presence of larger aggregates, consistent with immune complex formation. This bimodal size distribution confirms successful IC generation under the experimental conditions used.

Supplementary Table 1: Cytokines' Inter-group statistical comparison

Stimulation condition	Cytokine	Child group n = 15 *Median (min, max)*	Adult group n = 15 *Median (min, max)*	Elderly group n = 8 *Median (min, max)*	Comparison between child, adult and elderly groups *P value*			
					**K-W**	**Multiple comparisons**		
						**C vs A**	**C vs E**	**A vs E**
NS	TNF-α	178 (6 ; 814)	8 (3 ; 2224)	895 (153 ; 4593)	**0.0003**	0.12	0.09	**0.0002**
	IL1-α	18 (7 ; 38)	5 (3 ;91)	82 (14 ; 210)	**0.018**	0.45	0.37	**0.01**
	IFN-γ	31 (1 ; 175)	3 (0 ; 2 450)	44.50 (7.00 ; 1 699.00)	**0.020**	-	-	-
	IFN-α	1 (0.00 ; 135.00)	0 (0.00 ; 48.50)	1.50 (0.00 ; 355)	0.25	-	-	
	IP-10	21 (5 ; 133)	28 (2 ; 113)	25 (4.00 ; 112)	0.88	-	-	-
	IL-6	1230 (41 ; 5825)	1002 (17 ; 5684.5)	2561 (1128 ; 3844)	**0.042**	0.90	0.31	**0.04**
	IL-2	11 (2 ; 229)	8 (1 ; 344)	13 (5 ; 45)	0.167	-	-	-
	IL-10	176 (7 ; 1 432)	9 (4 ; 12 309.50)	662 (16; 2 589)	0.09	-	-	-
	IL1-RA	5 066 (26 ; 15 491)	2 770 (28.00 ; 17 808)	11 774.5 (2 797 ; 25 371)	**0.016**	-	-	-
IFN	TNF-α	338 (4; 5 036)	11 (6 ; 6 195.5)	1300.5 (243; 8 416)	**0.044**	-	-	-
	IL1-α	11 (3; 1 327)	5 (1 ; 9 035)	43.5 (11; 7 203)	0.06	-	-	-
	IFN-γ	23 (1; 286)	9 (2 ; 1 293)	187 (2; 1 716)	0.22	-	-	-
	IP-10	103 (13; 619)	153 (35 ; 1283)	216 (43; 375)	0.07	-	-	-
	IL-6	1057 (28; 5 807)	1 057 (74 ; 7 762)	2353 (641; 4 510)	0.49			
	IL-2	17 (11; 54)	17 (5 ; 62)	22 (14; 63)	0.50	-	-	-
	IL-10	22 (5; 1 851)	22 (9 ; 13 674.5)	867.5 (173; 9 084)	0.06	-	-	-
	IL1-RA	6898 (11 ; 24 137)	6 898 (960 ; 51 986)	12 081.5 (2 005; 22 366)	0.53	-	-	-
ICs	TNF-α	736 (27 ; 4433)	334 (7 ;5276)	4571 (74; 6360)	**0.032**	0.99	0.08	**0.03**
	IL1-α	43 (6 ; 121)	14.5 (8 ; 206)	91 (13; 185)	0.1224	-	-	-
	IFN-γ	81 (11 ; 1814)	797.5 (6 ; 2159)	39 (10; 738)	**0.02**	0.16	0.99	**0.04**
	IFN-α	130 (13 ; 954)	730 (0 ;13501)	44.5 (1; 412)	**0.0008**	**0.02**	0.67	**0.001**
	IP-10	81 (15; 999)	678 (138 ;1343)	33.5 (5; 446)	**0.0001**	**0.004**	0.87	**0.0003**
	IL-6	1846 (31;7328)	3065 (43 ; 5245)	1777 (833;6234)	0.45	-	-	-
	IL-2	36.5 (15 ;126)	81.5 (13;178)	16.5 (8; 44)	**0.002**	0.17	0.24	**0.002**
	IL-10	305 (39; 3776)	225.6 (6;15377.5)	1641 (87; 2917)	0.31	-	-	-
	IL1-RA	12423	17492	12499	0.53	-	-	-

		(19; 61848)	(2168.5 ;35769)	(4383; 30338)				
LPS	TNF-α	384<br>(41 ; 6462)	380<br>(27 ; 5794)	580<br>(7 ; 6603)	0.87	-	-	-
	IL1-α	71<br>(0 ; 228)	38.5<br>(10 ;152)	30<br>(1; 213)	0.77	-	-	-
	IFN-γ	37.50<br>(7 ; 577)	11<br>(0 ; 983)	226<br>(10 ; 1 151)	0.06	-	-	-
	IFN-α	7.50<br>(1 ; 37)	1<br>(1 ; 560)	14<br>(2 ; 441)	0.17	-	-	-
	IP-10	28<br>(7 ; 226)	9<br>(2 ; 542.5)	36<br>(6 ; 84)	0.19	-	-	-
	IL-6	2855<br>(1 ; 41700)	3413<br>(889 ; 6877.5)	2122<br>(7 ; 4806)	0.47	-	-	-
	IL-2	16<br>(9 ; 106)	11<br>(8 ; 45)	21<br>(11 ; 38)	0.07	-	-	-
	IL-10	1621<br>(56 ; 11679)	3094<br>(235 ;7061)	934<br>(488 ; 2128)	0.39	-	-	-
	IL1-RA	13473<br>(26 ; 51336)	14682<br>(4148 ;33486)	13783.00<br>(2038.00 ;<br>25661.00)	0.59	-	-	-

K-W=Kruskal Wallis Test, C= Child, A=Adult, E=Elderly
